# Importance of carbapenem-resistant Enterobacteriaceae screening to prevent transmission within an acute-care hospital

**DOI:** 10.1017/ash.2023.355

**Published:** 2023-09-29

**Authors:** Meghan Hudziec, Sarah Totten, Larissa Pisney, Cara Faliano

## Abstract

**Background:** Carbapenem-resistant Enterobacteriaceae (CRE) present a serious public health risk because they are transmissible within the acute-care hospital setting, and they are associated with significant morbidity and mortality. Timely identification of CRE among hospitalized patients is essential to ensure that appropriate infection prevention measures are enforced to prevent transmission events. In 2022, 9 index CRE cases (5 Klebsiella pneumoniae carbapenemase (KPC)–producing and 4 New Delhi Metallo-β-lactamase (NDM)–producing cases) were identified within the University of Colorado Hospital (UCH) inpatient population. In response to index case identification, tracing was performed to identify patients with an epidemiologic link for targeted CRE screening to detect asymptomatic CRE carriage. **Methods:** In total, 645 patients were screened allowing for timely identification of CRE colonization within 6 patients (3-KPC; 1-OXA-48; 1-NDM; 1-KPC/OXA-48). Secondary case identification elicited additional evaluation of service team and mobile-device crossover between positive patients, as well as primary and ancillary treatment locations. **Results:** Investigations revealed 3 possible transmission events in 0.47% of the total screened population. Identification of secondary CRE cases prompted additional testing of exposed patients performed at 7-day intervals to capture a 21-day colonization period. In total, 95 additional patients were screened for CRE during secondary and tertiary CRE screening events. **Discussion:** Nursing staff collaboration and engagement were critical to achieving a high rate of compliance with CRE screening activities, not only collecting screening specimens but also providing explanation and reassurance to patients. Due to this partnership and diligence, UCH was able to achieve 77% compliance with initial CRE screening events. Secondary and tertiary CRE screening revealed testing compliance of 83% and 69%, respectively. To further reduce the risk of CRE transmission within hospitalized patients, UCH has implemented an enhanced cleaning process for high-risk patient rooms, which includes patients infected or asymptomatically colonized with CRE. This enhanced process is prompted based on CRE or infection status as documented in the electronic medical record (EMR), and it initiates a mandatory 2-phase cleaning process. Future plans include environmental testing audits to validate room decontamination and leveraging the EMR to capture pertinent healthcare and travel histories. Active engagement with public health partners will be pursued to enable molecular testing of high-risk touch points. **Conclusions:** Patient screening, enhanced decontamination, and monitoring activities are key elements to effectively prevent the spread of CRE within vulnerable patient populations and must be continuously evaluated for improvement opportunities.

**Disclosures:** None

